# Doxorubicin-induced cardiotoxicity in adult Indian patients on chemotherapy

**DOI:** 10.4103/0971-5851.56329

**Published:** 2009

**Authors:** Navin Khattry, Pankaj Malhotra, Anil Grover, Suresh C Sharma, Subhash Varma

**Affiliations:** *Departments of Internal Medicine, Postgraduate Institute of Medical Education and Research, Chandigarh, India*; 2 *Department of Cardiology, Postgraduate Institute of Medical Education and Research, Chandigarh, India*; 3 *Department of Radiotherapy, Postgraduate Institute of Medical Education and Research, Chandigarh, India*

**Keywords:** *Doxurubicin*, *cardiotoxicity*, *echocardiographic monitoring*

## Abstract

**Background:**

Doxorubicin-induced cardiotoxicity is widely known to occur at cumulative doses exceeding 450 mg/m^2^. However, very few studies have reported incidence of cardiac dysfunction in patients on chemotherapy with lower cumulative doses. To the best of our knowledge, there is no study carried out so far that has reported the incidence of cardiac dysfunction in adult Indian patients receiving doxorubicin. This study was undertaken to determine the incidence of doxorubicin-induced cardiotoxicity by serial resting echocardiography in patients on chemotherapy and identify risk factors associated with cardiotoxicity.

**Materials and Methods:**

Patients that were started on doxorubicin-based chemotherapy in the period from January 2000 to June 2001 and had completed at least 300 mg/m^2^ cumulative dose were taken in the study. Electrocardiography, chest X-ray and echocardiography were done at baseline, at 300 mg/m^2^ and at 450 mg/m^2^ cumulative doses of doxorubicin. All patients were evaluated for the presence of the following risk factors: Age>70 years, female sex, preexisting cardiac disease, hypertension, chest wall irradiation, body mass index (BMI)<20 kg/m^2^ , Karnofsky performance status, combination chemotherapy with cyclophosphamide and presence of liver disease. Subclinical cardiac dysfunction was defined as ejection fraction fall greater than 10% on follow-up echocardiography.

**Results:**

Thirty patients satisfied the criterion for being considered for evaluation. One (3%) patient developed congestive cardiac failure, while 8 (27%) patients developed subclinical cardiac dysfunction. Concomitant use of cyclophosphamide significantly increased the risk of cardiac dysfunction (*P* = 0.048), while low BMI (<20 kg/m^2^) and preexisting cardiac disease showed a trend towards increased risk of cardiac dysfunction (*P* = 0.07 for both).

**Conclusion:**

Twenty-seven percent of the patients developed subclinical cardiac dysfunction in the cumulative dose range of 300-450 mg/m^2^ . This entails regular monitoring for cardiac dysfunction by echocardiography during treatment.

## INTRODUCTION

Anthracyclines represent some of the most commonly used anticancer drugs. Major side effects associated with anthracycline use are bone marrow suppression and cardiac toxicity. Cardiac toxicity is accentuated by increasing age (more than 70 years), female sex, combination chemotherapy, mediastinal radiation, previous cardiac disease, hypertension, liver disease and whole body hypothermia.[1]

Anthracyclines are associated with both acute and chronic cardiotoxicity. Chronic cardiotoxicity in the form of congestive cardiac failure is dose dependent and occurs 4-8 weeks after last anthracycline dose, though it may occur during treatment or years later. [2] Treatment with doxorubicin may necessitate lifelong cardiac monitoring. The exact causal mechanism of anthracycline-induced cardiac toxicity remains unclear, but most studies indicate that free radicals are involved.[1] Clinical parameters [3] and electrocardiographic [4] changes are not sensitive and specific in detecting cardiotoxicity. Endomyocardial biopsy, [5] though gold standard for detecting anthracycline-induced cardiotoxicity, cannot be employed routinely. Echocardiography [6] and radionuclide [7] imaging remain good noninvasive techniques to study effects on cardiac function. Though radionuclide studies have been reported to be more sensitive in detecting systolic and diastolic dysfunction, they expose patients to ionizing radiation [8] and are not widely available. Echocardiography is more easily available, is not dependent on the availability of radioisotopes and is less costly.

There are very few studies[9–11] reporting cardiac dysfunction in patients while still on chemotherapy. Though the risk of cardiac dysfunction increases steeply after a cumulative dose of 450-550 mg/m^2^ of doxorubicin is reached, it is not known whether lower cumulative doses also lead to significant cardiac dysfunction warranting very close monitoring.

This study was planned as a preliminary investigation to see whether echocardiography can detect cardiac dysfunction in patients on treatment with doxorubicin and also if there are any identifiable risk factors for cardiac toxicity in Indian patients.

## MATERIALS AND METHODS

All newly diagnosed patients aged more than 15 years who were administered doxorubicin for the first time and who gave informed consent were enrolled in the study. All patients were questioned for recent history of hypertension, mediastinal or chest wall irradiation and cardiac or liver disease. Body mass index (BMI) and Karnofsky performance status of all patients were recorded at baseline. A detailed examination of cardiovascular system was done at baseline and at each follow-up. Renal function tests, liver function tests, 12-lead ECG, chest X-ray and echocardiography were done at baseline, at 300 mg/m^2^ and at 400-450 mg/m^2^ cumulative doses of doxorubicin. During echocardiography, fractional shortening and ejection fraction were evaluated, besides other routine parameters. Fractional shortening was calculated by the formula

$\frac{{\mathrm{LVIDd}}^{*}-{\mathrm{LVIDs}}^{\delta }}{\mathrm{LVIDd}}\mathrm{\times 100}$

[*01LVIDd- Left ventricular internal diameter in diastole

^δ^ LVIDs- Left ventricular internal diameter in systole],

while ejection fraction was calculated by M-mode and modified Simpson′s formula. In order to reduce inter-observer variability, echocardiography was performed by the same physician for each patient at different time points. Intra-observer variability was reduced by taking the mean of three readings during each echocardiography.

All patients included in the study had left ventricular ejection fraction (LVEF) greater than 50% at baseline. Patients with history of coronary artery disease who had regional wall-motion abnormalities at baseline echocardiography or had an acute coronary syndrome within 1 year of cancer diagnosis were excluded from the study.

This study was approved by the ethics committee of the institute.

### Statistical analysis

Subclinical cardiac dysfunction was defined as fall of ejection fraction >10% during follow-up echocardiography at 300 mg/m^2^ and/or 400-450 mg/m^2^ . This was based on the published guidelines on monitoring for doxorubicin-induced cardiotoxcity.[6,12] Role of various risk factors was analyzed by using chi-square test and unpaired *t* test. The risk factors studied were age >70 years, female sex, presence of preexisting cardiac disease, hypertension, chest wall or mediastinal irradiation, BMI < 20 kg/m^2^ , Karnofsky performance status, combination chemotherapy with cyclophosphamide and presence of liver disease. Paired *t* test was used to compare various quantitative clinical parameters recorded at baseline, at 300 mg/m^2^ and 450 mg/m^2^ doses of doxorubicin. Multiple logistic regression analysis was done to analyze further the significance of individual risk factors.

## RESULTS

Thirty-eight patients were enrolled in the study, from January 2000 to June 2001. Eight patients were excluded as they did not receive full course of chemotherapy due to progression of disease. Thirty patients received at least 300 mg/m^2^ of doxorubicin, while 14 patients received more than 400 mg/m^2^ cumulative dose of doxorubicin. The baseline characteristics of 30 patients are shown in Table 1.

**Table 1:** Baseline characteristics of patients

Parameter	Mean (SD)	Range
Age (yrs)	44.83 ± 14.2	(16-72)
BMI (kg/m^2^)	23.36 ± 4.3	(17-35)
No. of cycles of chemotherapy received	7.63 ± 1.7	(6-10)
Cumulative dose of doxorubicin (mg/m^2^)	372.3 ± 79.4	(300-500)
Baseline heart rate	90.73 ± 11.2	(72-116)
Baseline SBP (mm Hg)	134.6 ± 11.9	(110-160)
Baseline DBP (mm Hg)	82.93 ± 9.4	(70-100)
Baseline EF	63.63 ± 7.04	(50-77)
Baseline FS	35.12 ± 6.04	(25-48)
BSA (m^2^)	1.6 ± 0.17	(1.3-2.1)
KPS	71.67 ± 10.9	(50-90)

BMI = Body mass index; BSA = Body surface area; SBP = Systolic blood pressure;

DBP = Diastolic blood pressure; EF = Ejection fraction; FS = Fractional shortening;

KPS - Karnofsky performance status; SD = Standard deviation

**Table 2:** Diagnosis and schedule of chemotherapy in patients evaluated for doxorubicin-induced cardiac dysfunction

Diagnosis	No. of pts. (*n* = 30)	Chemotherapy schedule	Dosing intervals	No. of cycles of chemotherapy
Non-Hodgkin's lymphoma	15 (50%)	Cy – 800 mg/m^2^	3 weekly	6-10
		Adr – 50 mg/m^2^		
		VCR – 1.4 mg/m^2^		
		Prn – 60 mg/m^2^		
Hodgkin's disease	5 (16.6%)	Adr – 25 mg/m^2^	4 weekly	6-8
		Blm – 10 U/m^2^ D1, D15		
		VBl – 6 mg/m^2^		
		DCZ – 375 mg/m^2^		
Breast carcinoma	4 (13.3%)	Cy – 600 mg/m^2^	3 weekly	6
		Adr – 50 mg/m^2^		
		5FU – 600 mg/m^2^		
Multiple myeloma	2 (6.67%)	VCR – 0.4 mg	3 weekly	6-8
		Adr – 9 mg/m^2^ D1-D4		
		Dex = 40 mg		
Gastric carcinoma	2 (6.67%)	5FU – 600 mg/m^2^	3 weekly	6
		Adr – 50 mg/m^2^		
		MMC – 6 mg/m^2^		
Hepatocellular	1 (3.33%)	5FU – 600 mg/m^2^	3 weekly	6
carcinoma		Adr – 50 mg/m^2^		
		MMC – 6 mg/m^2^		
Uterine	1 (3.33%)	Cy – 600 mg/m^2^	3 weekly	6
leiomyosarcoma		VCR – 1.4 mg/m^2^		
		Adr – 50 mg/m^2^		

Cy = Cyclophosphamide; Adr = Adriamycin; 5FU = 5 fluorouracil; Blm = Bleomycin; VCR = Vincristine; Dex = Dexamethasone; Prn = Prednisolone; MMC = Mitomycin C

DCZ = Dacarbazine, D1 = Day 1; D4 = Day 4; D15 = Day 15

**Table 3:** Prevalence of various risk factors among patients evaluated for cardiac dysfunction

Risk factors	No. of patients (%) (*n* = 30)
Hypertension	9 (30)
Mediastinal/chest wall irradiation	4 (13.33)
Heart disease	6 (20)
Combination therapy with cyclophosphamide	20 (66.67)
Age > 70 yrs	3 (10)
Liver disease	0 (0)
BMI ≤ 20 kg/m2	8 (26.6%)

The chemotherapeutic regimens used according to different diagnoses are shown in Table 2. Twenty (66.7%) patients received regimens that included both cyclophosphamide and adriamycin. The most common dose of adriamycin used was 50 mg/m^2^ per cycle of chemotherapy. The schedule of administration of doxorubicin was similar in all patients. All patients received intravenous boluses of doxorubicin over 10 minutes. Three patients had postponement of cycle of chemotherapy by 1 week due to neutropenia. However, all 30 patients completed the designated number of cycles of chemotherapy according to their protocol.

The prevalence of various risk factors is shown in Table 3. The most commonly associated risk factor was the concomitant use of cyclophosphamide (66.7%).

Nine (30%) patients had greater than 10% fall in LVEF as indicated in follow-up echocardiography (group A). Twenty-one patients had either no change or less than 10% fall in LVEF (group B). In group A, 1 (3%) patient developed congestive heart failure after receiving 450 mg/m^2^ cumulative dose of doxorubicin, while 8 (27%) patients developed subclinical cardiac dysfunction at 300 mg/m^2^ (4 patients) or 400-450 mg/m^2^ (4 patients) cumulative dose. There was no significant difference in systolic and diastolic blood pressure in groups A and B ‒ at baseline, 300 mg/m^2^ and 400-450 mg/m^2^ cumulative dose of doxorubicin as analyzed by paired *t* test. Similarly no significant changes were seen in ECG and chest x-ray of patients at above dose levels. No patient developed acute cardiac dysfunction after any dose of doxorubicin.

Age >70 years, sex, hypertension, concomitant or previous use of mediastinal/chest wall irradiation and Karnofsky performance status did not correlate significantly to the development of cardiac dysfunction (*P* >0.1). However, multiple logistic regression analysis showed that concomitant chemotherapy with cyclophosphamide significantly correlated with increased incidence of doxorubicin-induced cardiotoxicity (regression coefficient = 3.22, *P* =0.048). Similarly lower BMI (< 20 kg/m^2^ ) and presence of associated heart disease correlated with increased incidence of subclinical cardiotoxicity (regression coefficient = −4.321, *P* = 0.07; *r* = 4.09, *P* = 0.07), though they did not reach statistical significance.

## DISCUSSION

It is a well-known fact that incidence of cardiac dysfunction rises steeply if cumulative dose of doxorubicin exceeds 550 mg/m^2^ . However, very few studies have evaluated development of asymptomatic cardiac dysfunction in the lower cumulative dose range. The present study was aimed to find the incidence of both subclinical and clinical cardiac dysfunction at lower cumulative dose range, viz., 300 to 450 mg/m^2^ , by serial echocardiographic measurement and identify any known risk factors associated with cardiac dysfunction.

The incidence of clinically diagnosed doxorubicin-induced congestive heart failure was 3% in our study and is in general agreement with the range of 0.4% to 9% reported by others.[13,14] Though the incidence of congestive cardiac failure was low, incidence of subclinical cardiac dysfunction in our study was high, viz., 27% (8 of the 30 patients). This observation is consistent with studies that have evaluated subclinical cardiac dysfunction. Palmeri *et al,*[9] in their group of 48 patients that received a mean dose of doxorubicin of 338 mg/m^2^ found that 63% of their patients had some fall in LVEF as measured by rest and exercise radionuclide angiography. Similarly Dresdale *et al.,*[10] in 87 asymptomatic patients that received >430 mg/m^2^ of doxorubicin found abnormal LVEF at rest by radionuclide angiogram in 21% of patients. Exercise studies in their study identified an additional 31% of patients. Mohta *et al.,*[15] in pediatric patients observed that 30% of the patients had significant cardiac dysfunction on echocardiographic evaluation at a mean cumulative dose of 365 mg/m^2^ . Similarly Agarwala *et al.,*[11] observed that 40% of children undergoing doxorubicin-based chemotherapy developed subclinical cardiac dysfunction at a cumulative dose of 180-200 mg/m^2^ .

Cumulative dose of doxorubicin is the single most important determinant of cardiac toxicity. Lefrak *et al.,*[16] in their study found that incidence of congestive cardiac failure rose to unacceptably high levels when cumulative dose of drug exceeded 550 mg/m^2^ , viz., from 4% at 500-550 mg/m^2^ to 18% at 551-600 mg/m^2^ and to 36% at a dose of 601 mg/m^2^ or more. Von Hoff *et al.,*[17] found that cumulative probability of developing drug-induced heart failure was 0.03 at 400 mg/m^2^ , 0.07 at 550 mg/m^2^ and 0.18 at 700 mg/m^2^ . In the present study, at 300 mg/m^2^ cumulative dose of doxorubicin, 16% developed subclinical cardiac dysfunction; and at 450 mg/m^2^ , 28.8% of patients develop cardiac dysfunction. This difference did not reach statistical significance possibly due to the small size of the study population (χ^2^=0.26, P >0.1).

The present study found statistically significant association of concomitant use of doxorubicin and cyclophosphamide with cardiac dysfunction (regression coefficient = 3.22, *P* = 0.048). Similar inference was drawn by Minow *et al.* [13] and von Hoff *et al.*,[17] in their studies, and it is now standard practice to use lower cumulative dose ceiling in patients exposed to both cyclophosphamide and doxorubicin.

Malnutrition has also been identified as a risk factor for doxorubicin-induced cardiotoxicity. A study by Obama *et al.,*[18] found that cardiac toxicity was more in those children that were malnourished (P < 0.05). Similarly Mohta *et al.,*[15] in Indian pediatric cancer patients observed that children that developed cardiac dysfunction had significantly lower height and weight for age compared to those that remained normal. Though we did not do a formal nutritional assessment in our patients, we analyzed the incidence of cardiac dysfunction in patients with lower BMI (< 20 kg/m^2^ ) as a surrogate marker of nutritional status. Patients with BMI < 20 kg/m^2^ were shown in our study to have increased risk of cardiac dysfunction, though the association did not reach statistically significant level, probably due to the small sample size (regression coefficient = −0.432, *P* = 0.07). How poor nutritional status affects cardiotoxicity is not clear, though it has been reported that micronutrients like carnitine, [19] selenium [20] and vitamin E [21] have ameliorated cardiotoxicity of anthracyclines.

In our study, 6 (20%) patients had medical history of coronary artery disease. All 6 patients had LVEF greater than 50% at baseline with no regional wall motion abnormalities in echocardiogram. Two out of 9 patients with doxorubicin-induced cardiac dysfunction had coronary artery disease. Multiple logistic regression analysis showed that cardiac disease as a risk factor almost reached statistical significance in contributing to doxorubicin-induced cardiotoxicity in our patients (regression coefficient = 4.088, *P* = 0.07). The results are similar to those in the studies by von Hoff *et al.*[17] and Minow *et al.,*[13] who found that patients with preexisting cardiac disease had increased probability of developing cardiac dysfunction due to doxorubicin.

The present study due to its small sample size did not find hypertension, advanced age, receiving mediastinal irradiation, sex and performance status to be risk factors for cardiotoxicity.

The high incidence of subclinical cardiac dysfunction in our patients reinforces the need for strict monitoring of patients on doxorubicin during treatment.

In conclusions, the present study suggests that subclinical cardiac dysfunction due to doxorubicin is high in the cumulative dose range of 300-450 mg/m^2^ . This entails regular monitoring for cardiac dysfunction by echocardiography during treatment.
